# Supplementary material for: Maternal Consumption of a Diet Rich in Maillard Reaction Products Accelerates Neurodevelopment in F1 and Sex-Dependently Affects Behavioral Phenotype in F2 Rat Offspring
Source: Foods. 2019 May 17;8(5):168. doi: 10.3390/foods8050168 (PMC6560437; doi:10.3390/foods8050168)
Supplement: Supplementary file 1 [file foods-08-00168-s001.pdf]

Supplementary Table S1. F0 dams: Behavioral tests results

	CTRL	AGE-RD	p
**Open field test**			
Time spent in central zone (%)	2.5 ± 1.6	2.9 ± 3.4	0.790
Distance travelled (cm)	1860 ± 419	1573 ± 246	0.133
Velocity (cm/s)	6.2 ± 1.4	5.2 ± 0.8	0.133
**Novel object recognition test**			
Exploration ratio (%)	49.5± 23.1	54.3 ± 11.6	0.654
**Light-dark test**			
Time spent in light arm (%)	73.4± 9.5	71.5 ± 19.5	0.834
**Elevated plus maze**			
Preference of open arm entries (%)	35.3± 14.3	34.6 ± 23.8	0.950
Time spent in open arms (%)	40.5± 19.7	40.7 ± 27.1	0.992
**Splash test**			
Latency (s)	45 ± 23	181 ± 106	**0.008**
Grooming (s)	108± 51	48 ± 81	0.167

CTRL, dams consuming standard chow; AGE-RD, dams fed diet rich in advanced glycation end products over 10 weeks (four weeks before mating, three weeks during pregnancy, three weeks during lactation).

Supplementary Table S2. Percentage difference in the area under the curve of the presence of reflex manifestation of offspring of F0 dams consuming advanced glycation end products rich diet versus those on the control diet.

	F1 females	F1 males	F2 females	F2 males
Ear twitch reflex	-0.6	2.6	3.7	6.13
Forelimb placing	-4.0	11.6	0.1	10.4
Gait	4.7	-1.0	-7.0	7.9
Air righting	14.1	1.8	3.7	2.4

F1, first generation offspring; F2, second generation offspring; positive values indicate better performance in offspring of dams (F0) consuming diet rich in advanced glycation end products

Supplementary Table S3: Behavioral tests results in F1 and F2 generation.

G/Sex	CTRL$_{7w}$	CTRL$_{6M}$	CTRL$_{12M}$	AGE-RD$_{7w}$	AGE-RD$_{6M}$	AGE-RD$_{12M}$
**Open field test**						
Velocity (cm/s)						
F1/F	6.2±1.0	5.5±1.0	4.3±1.1	7.0±0.6	5.6±0.8	4.3±0.6
F2/F	5.9±1.1	5.3±0.8	4.9±1.3	5.2±1.3	5.0±1.5	4.1±1.4
F1/M	6.1±0.9	4.5±0.9	3.1±0.7	6.0±0.6	4.3±0.7	2.7±0.9
F2/M	5.4±0.8	3.6±0.8	3.1±1.0	4.2±1.2	4.2±1.0	2.9±0.7
F: Velocity of ambulation decreased by age ($F_{(2, 82)}$=44.9, p < 0.001), without significant effects of age*F0 diet (p = 0.454) or age*F0 diet*generation interaction (p = 0.397).<br>M: Velocity of ambulation decreased by age ($F_{(2, 85)}$=103.5, p < 0.001), without significant effects of age*F0 diet (p = 0.061). Age*F0 diet*generation interaction reached p = 0.018. In F2 generation ($F_{(2, 47)}$=26.4, p = 0.003). Compared with their CTRL counterparts, AGE-RD males ran more slowly in pubertal age (p = 0.015). CTRL males showed slower ambulation both in adulthood and at old age (p < 0.01; both) compared with puberty, AGE-RD offspring ran with lower speed only in old age (p = 0.018).						
**Light-dark test**						
Time in light chamber (%)						
F1/F	50.5±31.7	83.6±18.1	59.2±28.1	43.2±27.4	71.0±28.8	50.5±19.7
F2/F	51.7±27.9	81.9±20.5	95.1±7.2	59.2±22.1	67.3±25.0	69.4±29.6
F1/M	36.7±30.9	79.8±19.4	62.3±33.3	54.8±24.4	84.3±16.9	55.9±22.1
F2/M	35.9±25.8	61.3±32.1	71.7±35.7	42.0±36.8	69.9±21.7	77.0±30.8
With increasing age, offspring spent longer time in illuminated part of light-dark box, without any significant impact of age*F0 diet or age*F0 diet*generation interaction. F: $F_{(2, 85)}$=19.6, p < 0.001; age*F0 diet: p = 0.092; age*F0 diet*generation interaction: p = 0.159. M: $F_{(2, 85)}$=20.4, p < 0.00 1; age*F0 diet: p = 0.459; *F0 diet*generation interaction: p = 0.486.						
**Elevated plus maze**						
Open arm entries frequency (%)						
F1/F	50.7±12.9	51.7±14.8	24.9±24.0	49.9±14.0	45.4±17.9	27.7±9.1
F2/F	45.1±18.7	29.0±19.3	32.2±23.8	51.6±14.4	43.2±14.6	43.3±20.3
F1/M	45.2±12.9	34.0±18.2	16.5±15.8	47.5±18.7	49.6±24.2	30.9±25.7
F2/M	38.8±13.6	26.4±16.6	39.3±27.3	40.7±14.0	37.3±18.9	34.3±28.4
GLM indicated significance in open arms entries frequency (F: $F_{(2, 43)}$=29.2, p < 0.001; M: $F_{(2, 38)}$=14.3, p < 0.001), without a significant impact of age*F0 diet in either setting (p = 0.176, 0.770, 0.774 and 0.348, respectively).						
**Novel object recognition test**						
Exploration ratio (%)						
F1/F	64.1±16.2	53.7±14.7	54.9±8.8	54.1±17.7	57.1±13.2	56.2±9.9
F2/F	64.9±11.0	53.2±15.9	50.2±15.7	60.2±19.3	52.0±9.4	44.4±16.3
F1/M	61.5±21.4	52.4±20.8	36.5±24.5	53.9±12.8	48.3±13.9	55.4±22.4
F2/M	58.0±18.1	55.9±14.6	54.0±31.4	49.0±15.5	38.1±28.6	58.4±26.0
Repeated measures GLM indicated significant difference in a preference to sniff a novel object in females ($F_{(2, 100)}$=6.3, p = 0.003) but significant effect of age*F0 diet: p = 0.300 or age*F0 diet*generation interaction: p = 0.697 has not been confirmed. Neither of investigated factors affected significantly the exploration ratio in male offspring ($F_{(2, 76)}$=1.3, p = 0.286).						
**Splash test**						
Latency to groom (s)						
F1/F	82±77	111±79	167±111	84±75	127±66	77±57
F2/F	133±99	156±94	162±119	109±70	77±81	117±98
F1/M	174±960	160±94	96±68	150±96	140±65	69±57
F2/M	72±74	165±104	102±76	108±61	133±97	122±71

GLM indicated no significant difference in latency to groom (F: $F_{(2, 99)}$=1.6, p = 0.215; M: $F_{(2, 93)}$=0.61, p = 0.427).						
Grooming (s)						
F1/F	107±56	64±51	44±59	121±54	79±46	80±67
F2/F	44±39	63±53	51±55	57±37	105±60	95±61
F1/M	45±36	53±51	42±37	90±61	69±42	92±49
F2/M	60±40	38±45	58±43	65±42	80±55	86±41
GLM did not indicate a significant difference in grooming time (F: $F_{(2, 89)}$=0.83, p = 0.432; M: $F_{(2,91)}$=0.60, p = 0.545).						

G, generation; CTRL = F1 and F2 offspring of F0 dams fed control diet; F0/AGE-RD group = F1 and F2 offspring of F0 dams fed a diet rich in advanced glycation end-products; 7w = 7 weeks of age; 6m = 6 months of age; 12m = 12 months of age; F1, first generation offspring; F2, second generation offspring; F, female; M, male; GLM, general linear model with F0 diet (CTRL, AGE-RD), offspring age at testing (7w, 6m, 12m) and generation (F1, F2) entered as fixed variables; data are presented as mean ± standard deviation

Supplementary Figure S1. Mean day of the eye and auditory conduit opening maturation in offspring

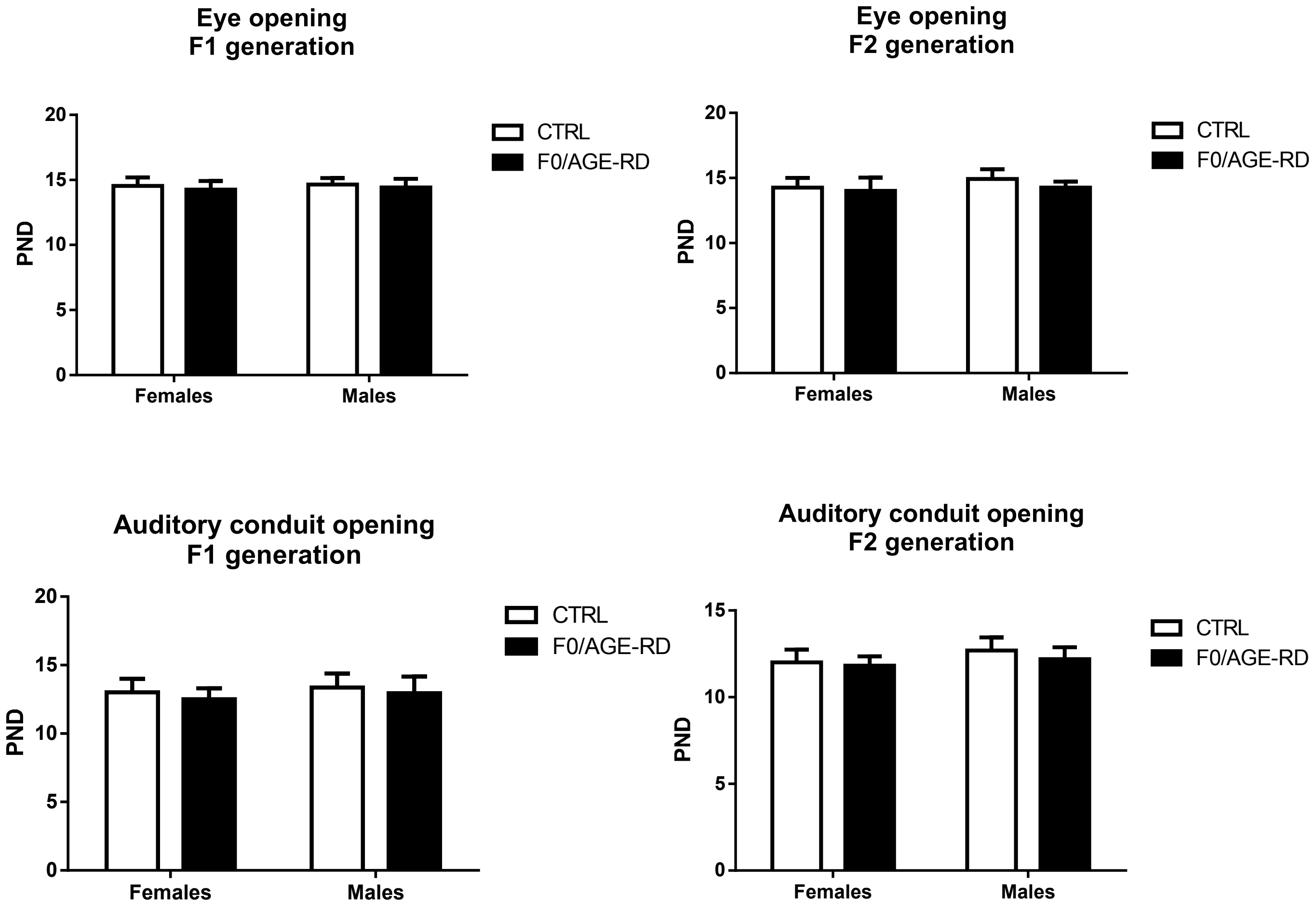

CTRL = F1 and F2 offspring of F0 dams fed control diet; F0/AGE-RD group = F1 and F2 offspring of F0 dams fed a diet rich in advanced glycation end-products; PND, postnatal day; data are presented as mean ± standard deviation

Supplementary Figure S2. Mean day of auditory startle reflex appearance in offspring

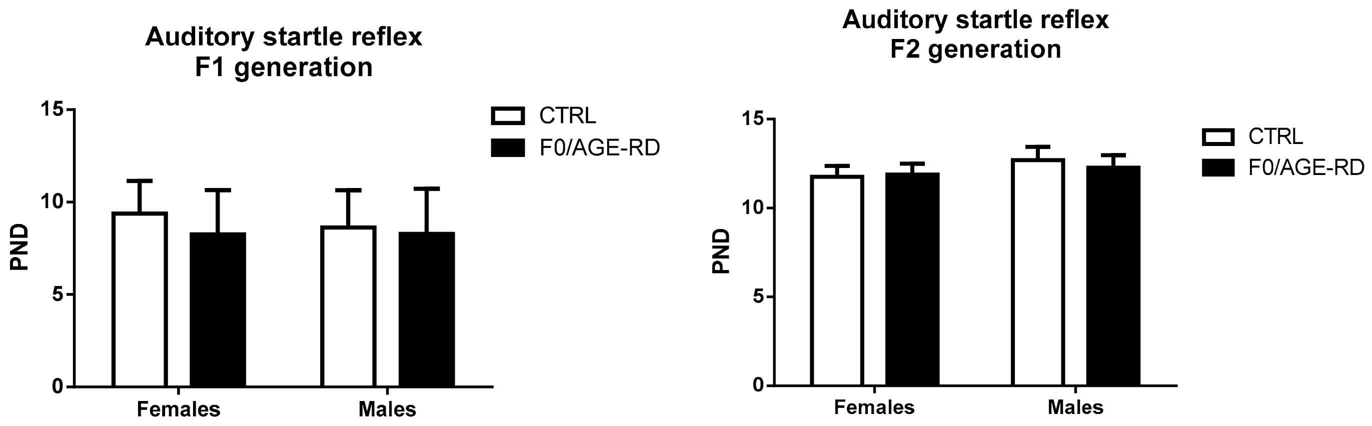

CTRL = F1 and F2 offspring of F0 dams fed control diet; F0/AGE-RD group = F1 and F2 offspring of F0 dams fed a diet rich in advanced glycation end-products; PND, postnatal day; data are presented as mean ± standard deviation

Supplementary Figure S3. Mean day of the eyelid reflex manifestation and incisor eruption in offspring

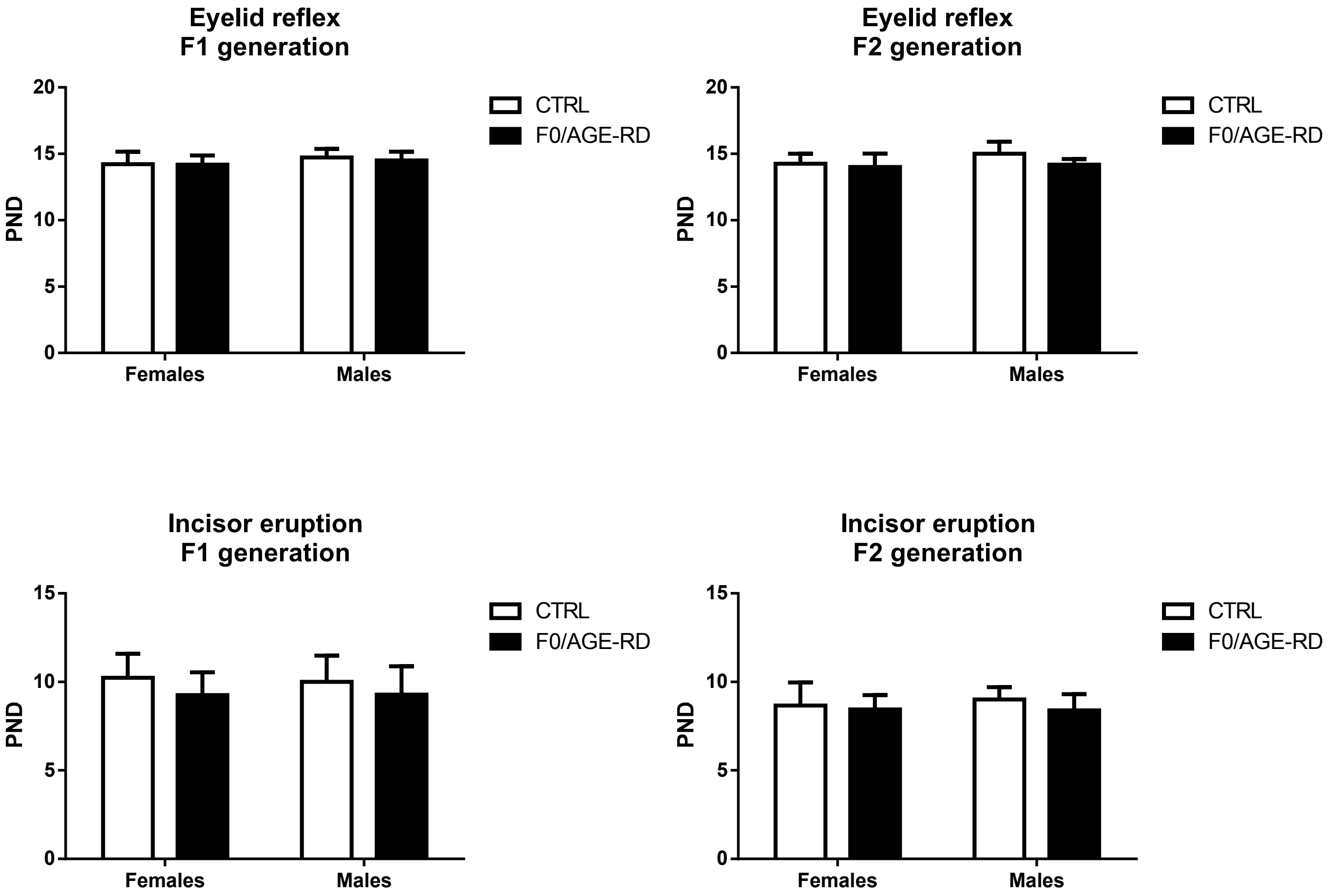

CTRL = F1 and F2 offspring of F0 dams fed control diet; F0/AGE-RD group = F1 and F2 offspring of F0 dams fed a diet rich in advanced glycation end-products; PND, postnatal day; data are presented as mean ± standard deviation

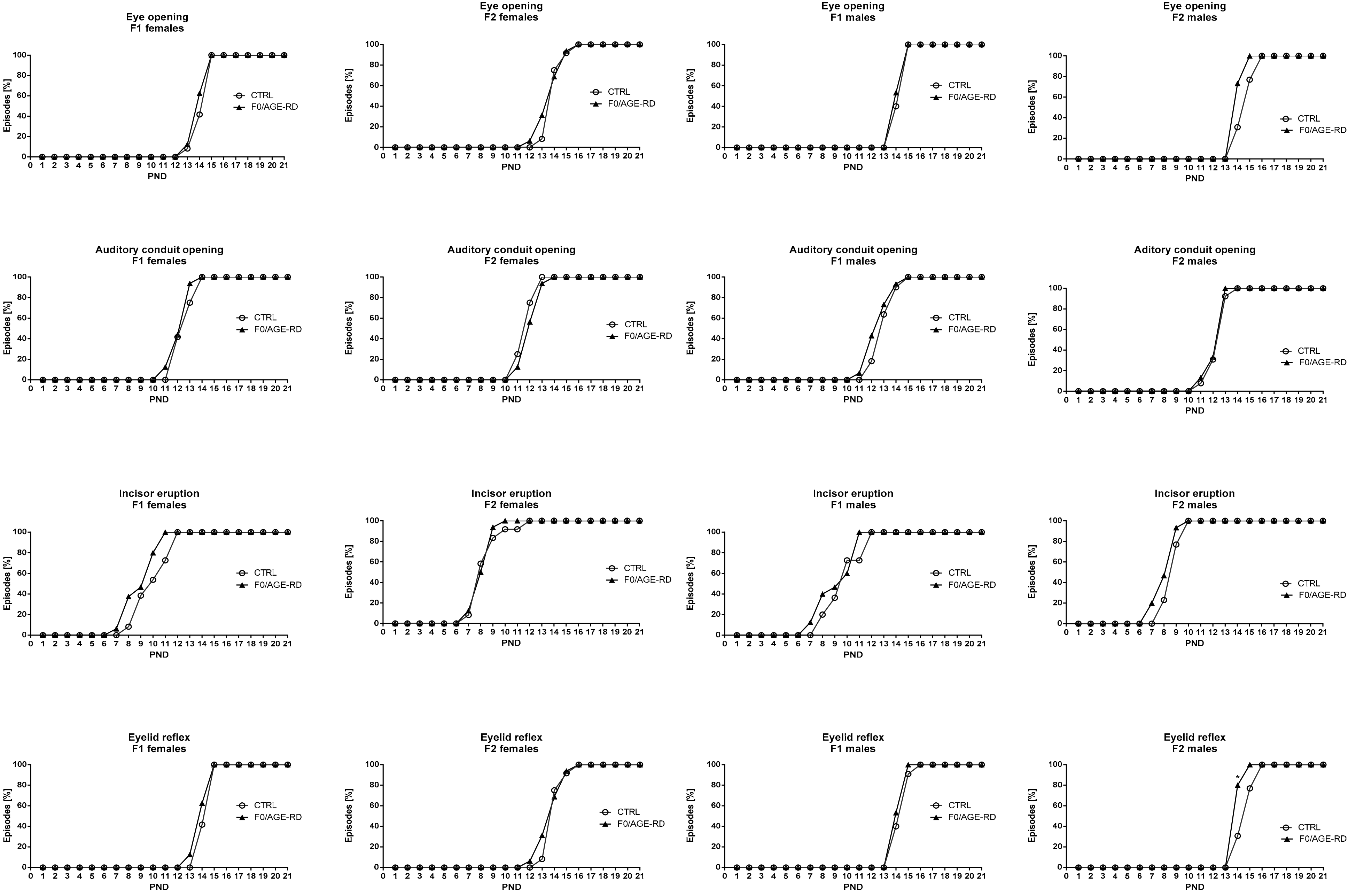

Supplementary Figure S4. Percentage of animals with developed motoric reflexes. * $p_{Chi} < 0.05$ (control - CTRL vs. advanced glycation end-products-rich diet - AGE-RD offspring)

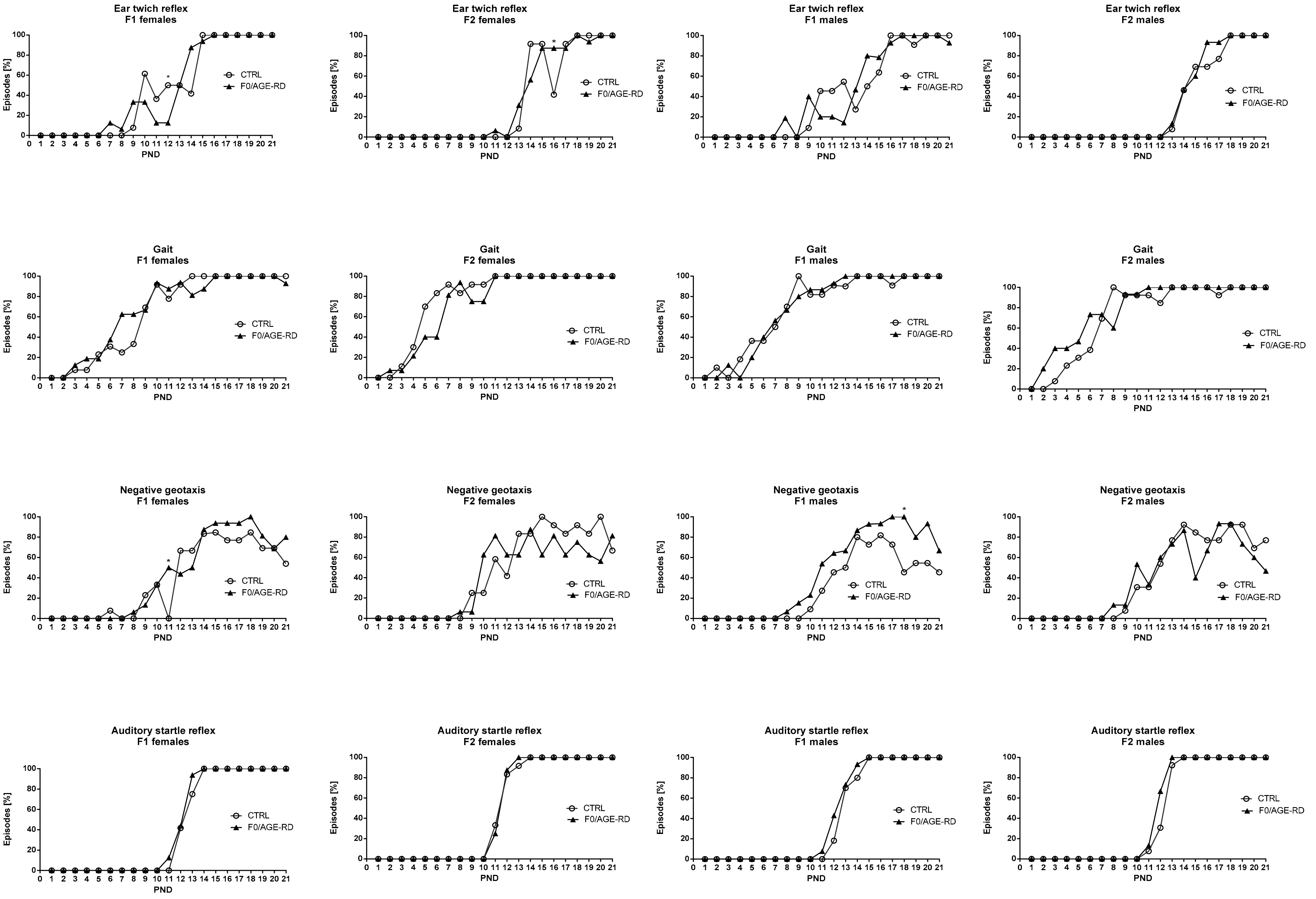

Supplementary Figure S5. Percentage of animals with developed motoric reflexes. * $p_{Chi} < 0.05$ (control - CTRL vs. advanced glycation end-products-rich diet - AGE-RD offspring)

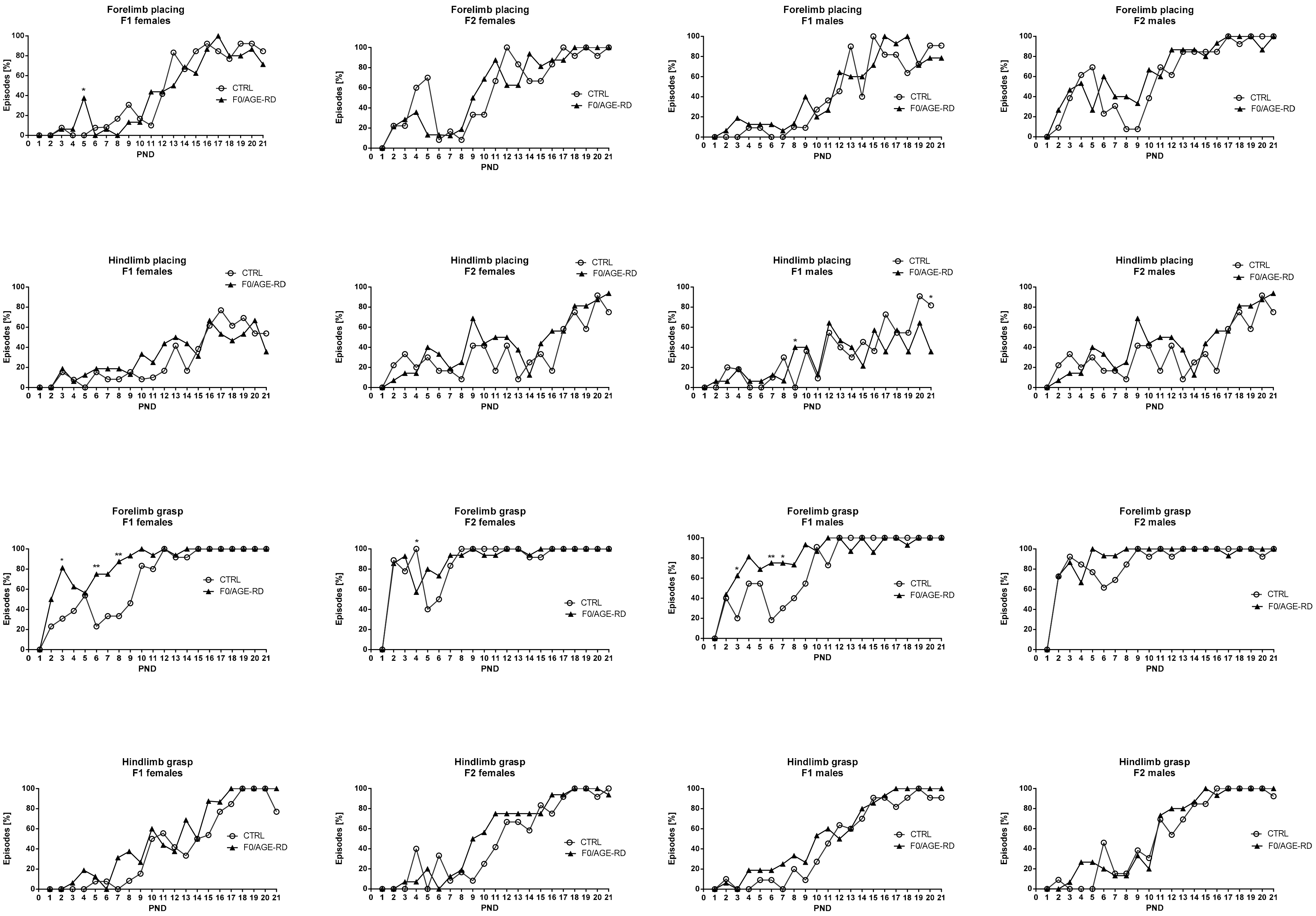

Supplementary Figure S6. Percentage of animals with developed motoric reflexes. * $p_{Chi} < 0.05$; ** $p_{Chi} < 0.01$ (control - CTRL vs. advanced glycation end-products-rich diet - AGE-RD offspring)

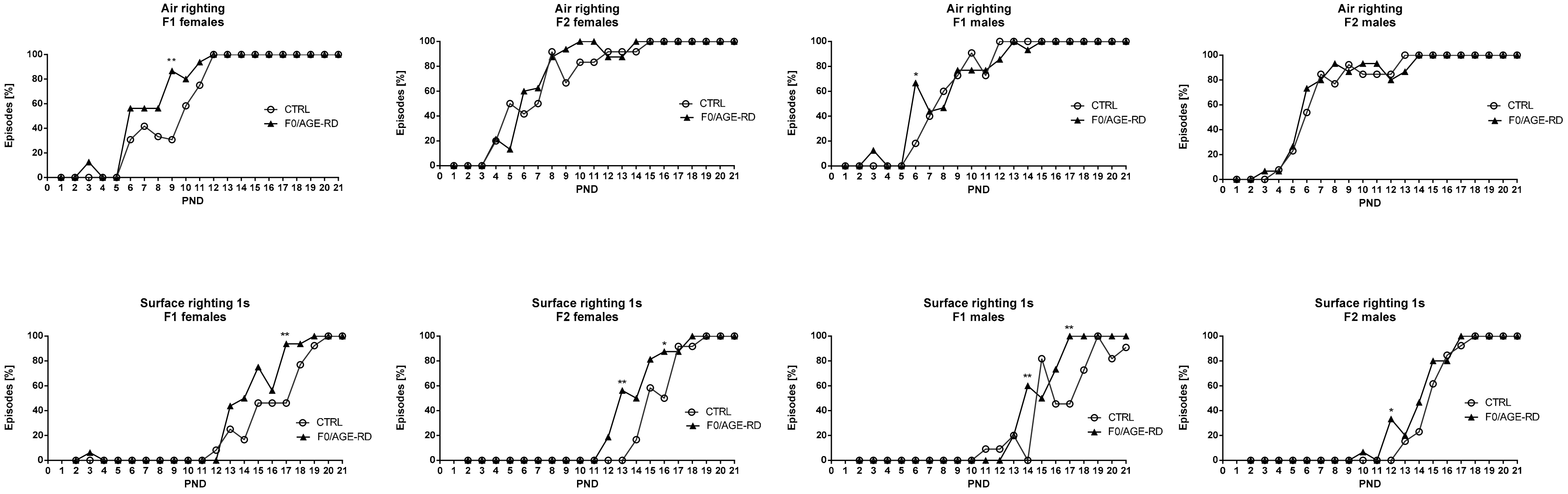

Supplementary Figure S7. Percentage of animals with developed motoric reflexes * $p_{Chi} < 0.05$; ** $p_{Chi} < 0.01$ (control - CTRL vs. advanced glycation end-products-rich diet - AGE-RD offspring)
